# Expression of Reversion-Inducing Cysteine-Rich Protein with Kazal Motifs (*RECK*) Gene and Its Regulation by miR200b in Ovarian Endometriosis

**DOI:** 10.3390/ijms252111594

**Published:** 2024-10-29

**Authors:** Agata Gozdz, Radosław B. Maksym, Aneta Ścieżyńska, Martin Götte, Claudine Kieda, Paweł K. Włodarski, Jacek Malejczyk

**Affiliations:** 1 Department of Histology and Embryology, Center of Biostructure Research, Medical University of Warsaw, ul. T. Chałubińskiego 5, 02-004 Warsaw, Poland; radoslaw.maksym@cmkp.edu.pl (R.B.M.); asciezynska@wum.edu.pl (A.Ś.); pawel.wlodarski@wum.edu.pl (P.K.W.); 2 1st Department of Obstetrics and Gynecology, Centre for Postgraduate Medical Education, ul. Żelazna 90, 01-004 Warsaw, Poland; 3 Center for Molecular Biophysics UPR 4301 CNRS, 45071 Orleans, France; claudine.kieda@cnrs-orleans.fr; 4 Laboratory of Molecular Oncology and Innovative Therapies, Military Institute of Medicine-National Research Institute, 04-141 Warsaw, Poland; 5 Department of Obstetrics and Gynecology, University Hospital Münster, Albert-Schweitzer-Campus 1, 48149 Münster, Germany; martin.goette@ukmuenster.de; 6 Cells-in-Motion Interfaculty Centre (CiMIC), University of Münster, 48149 Münster, Germany

**Keywords:** endometriosis, *RECK*, mir200, MMP, TIMP, matrix remodeling, adhesions, collagen, extracellular matrix, endometrial cyst, endometrioma

## Abstract

Endometriosis is a common chronic disorder characterized by the growth of endometrium-like tissue outside the uterine cavity. The disease is associated with chronic inflammation and pelvic pain and may have an impact on the patient’s fertility. The causative factors and pathophysiology of the disease are still poorly recognized. The dysregulation of the immune system, aberrant tissue remodeling, and angiogenesis contribute to the disease progression. In endometriosis patients, the proteins regulating the breakdown and reorganization of the connective tissue, e.g., collagenases, and other proteases, as well as their inhibitors, show an incorrect pattern of expression. Here, we report that the expression of reversion-inducing cysteine-rich protein with Kazal motifs (*RECK*), one of the inhibitors of connective tissue proteases, is elevated in endometrioma cysts as compared to normal endometrium from unaffected women. We also demonstrate a reduced level of miR200b in endometriotic tissue that correlates with *RECK* mRNA levels. Furthermore, we employ the 12Z cell line, derived from a peritoneal endometriotic lesion, and the Ishikawa cell line, originating from endometrial adenocarcinoma to identify *RECK* as a direct target of miR200b. The described effect of miR200b on *RECK*, together with the aberrant expression of both genes in endometrioma, may help to understand the role played by the tissue remodeling system in the pathogenesis of endometriosis.

## 1. Introduction

Endometriosis is a common chronic disorder affecting about 10% of women at a reproductive age. It is related to the presence and growth of endometrial tissue outside the uterine cavity, especially in the peritoneum and on the ovaries [1,2]. The disease is associated with chronic inflammation and manifests with pelvic pains and subfertility. The mechanisms leading to the formation of ectopic endometrial lesions are still a matter of dispute. The most accepted Sampson’s theory assumes that ectopic endometrial tissue foci are formed by shed endometrial cells, which get into the peritoneal cavity by retrograde menstruation [3,4]. The mechanisms responsible for the survival, implantation, and growth of endometriotic cells in the ectopic environment are still poorly recognized. It is speculated that it may be due to the hampered eradication of endometriotic cells by the cells of the local immune system [5,6,7]. Furthermore, it has been demonstrated that cells from endometriotic lesions are less susceptible to the induction of apoptotic cell death [8,9]. They also display an increased adhesiveness and invasiveness that may account for a higher rate of implantation [10,11,12,13]. Another factor that is crucial for the development and progression of endometriotic lesions is an increased induction of local angiogenesis [14,15,16]. 

Cell invasiveness and angiogenesis depend on the local release of matrix-degrading factors, in particular matrix metalloproteinases (MMPs) belonging to the zinc/calcium-dependent endopeptidases family [17,18,19,20]. Indeed, endometriosis has been reported to be associated with the increased production and release of a variety of MMPs, especially MMP-2 and MMP-9 [12,21,22,23]. The expression of MMPs may be regulated by many different factors, including proinflammatory cytokines and proangiogenic factors, as well as TGF-β [18,24,25,26,27]. The proteolytic activity of MMPs is also under the regulation of the tissue inhibitors of metalloproteinases (TIMPs) [19,20]. TIMPs have been reported to be expressed in endometriotic tissues, and there is a growing bulk of evidence that the MMP/TIMP balance plays a crucial role in the pathogenesis of endometriosis [12,21,28,29,30]. This balance is hypoxia-dependent and may be affected by the aberrant activation of the immune system in endometriosis patients [31,32]. 

In addition to typical members of the TIMP family, MMP inhibitors also include reversion-inducing cysteine-rich protein with Kazal motifs (*RECK*), a 971-amino acid glycosyl-phosphatidylinositol (GPI)-anchored membrane glycoprotein [33,34,35,36,37]. The human *RECK* gene is 87 kb long and contains 21 exons and 20 introns and is located on chromosome region 9p12-p12. *RECK* is a cysteine-rich glycoprotein displaying three serine protease inhibitor-like (SPI) domains, two epidermal growth factor-like repeats, and hydrophobic N- and C-terminal domains [32,33].

*RECK* has been recognized as an inhibitor of MMP-2, -9, and -14 as well as ADAM-10 and -17 metalloproteinases [36,38]. The latter activity may be involved in the regulation of some signaling pathways e.g., TNF release and Notch pathway activity [39,40,41]. Thus, *RECK* is considered an important regulator of extracellular matrix remodeling in the course of cell invasion and angiogenesis, as well as intercellular signaling [33,35,36,42]. The biological function of *RECK* strongly suggests that it may play a part in the pathogenesis of endometriosis. However, there are no published data on aberrant *RECK* expression or its regulation in endometriotic tissue. 

One of the possible modes of gene expression control occurs at the posttranscriptional level and depends on the action of microRNA. MicroRNAs (miRNAs, miRs) are short RNA species that recognize and bind specific sequences, e.g., seed sequences, located at the targeted mRNA molecules. MicroRNA binding in most cases leads to the degradation of a target mRNA or blocks its translation without mRNA degradation [43,44,45,46]. MicroRNAs control cell differentiation and survival, epithelial-to-mesenchymal transition, cell motility, and interaction with the immune system [43,44,45,46]. As the above processes have been shown to be disturbed in endometriosis patients and contribute to the disease progression [10,12,15,16], an increasing research effort is being put into identifying microRNAs’ role in endometriosis pathogenesis. Indeed, several microRNA types display an altered expression in endometriotic tissue, including species targeting genes regulating angiogenesis and connective tissue remodeling, like several members of the TIMPs family [47]. 

*RECK* mRNA was shown to be targeted by different microRNAs in several tissues and cell types [48,49,50,51,52,53,54,55,56,57,58,59,60,61]. However, there is no evidence of the involvement of microRNAs in the regulation of *RECK* mRNA levels in endometriosis. Therefore, the aim of the present study was (i) to compare the expression of *RECK* mRNA in samples from normal endometrium and endometriotic ovarian cysts, (ii) to examine the expression of miRNAs putatively involved in *RECK* mRNA regulation and their possible correlation, and (iii) to determine whether the investigated miRNAs regulate *RECK* mRNA expression in cell lines originating from the endometrium. 

## 2. Results

**Figure 1:**
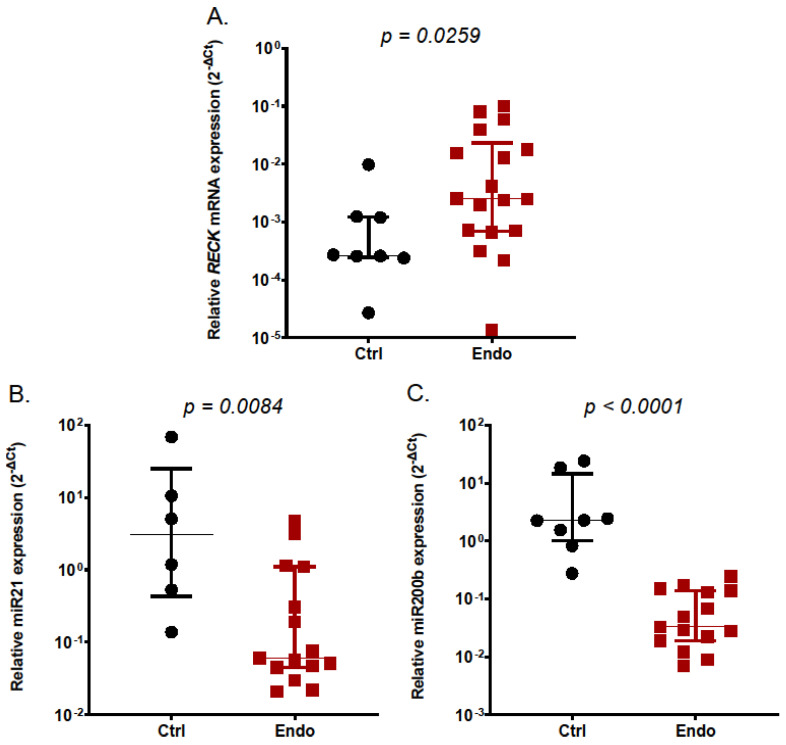
Expression of *RECK* mRNA (**A**), miR21 (**B**), and miR200b (**C**) in the endometrium from control subjects (Ctrl, *n* = 8) and ectopic endometriotic lesions (Endo, *n* = 14) in the luteal phase of the menstrual cycle. Expression of genes of interest was determined with qRT-PCR. Data are shown as scatterplots with the median and interquartile range. Statistical differences between groups were computed by the Mann–Whitney U-test.

**Figure 2:**
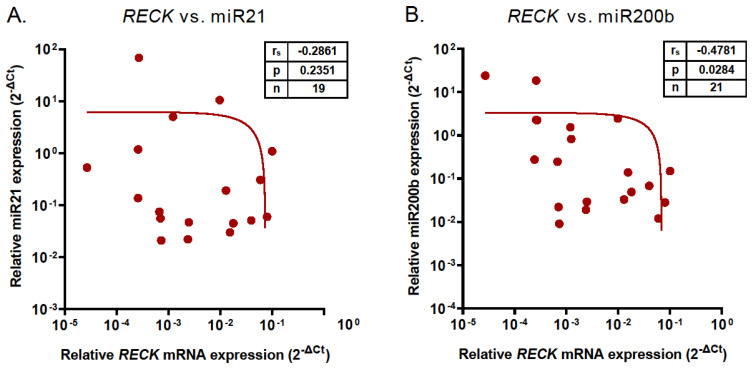
Correlation of miR21 (**A**) and miR200b (**B**) with *RECK* mRNA expression in pooled samples from eutopic control endometrium and endometriotic lesions. Insets show r_s_, *p* values, and number of samples (n).

To find out whether *RECK* mRNA expression is altered in endometriotic lesions, we compared its levels in eutopic endometrium from control women and ectopic endometrium from endometriotic cysts. As seen in Figure 1A, *RECK* expression was significantly higher (*p =* 0.0259) in endometriotic tissue than in control eutopic endometrium as determined by qRT-PCR. As *RECK* mRNA 3′UTR appears to be targeted by several microRNA species (Figure 2), we measured the level of miRNAs putatively regulating *RECK* levels in endometriotic and control tissue. We found that the expression of miR200b and miR21 was significantly downregulated in endometriotic tissue (Figure 1B,C). The levels of miR181a and miR182 remained unaffected.

These observations prompted us to investigate if there was any correlation between *RECK* mRNA and miR21 or miR200b levels. A Spearman correlation analysis revealed that the expression of *RECK* and miR200b genes was significantly inversely correlated (r_s_ = −0.4781, *p* = 0.0284, Figure 2A). There was no significant correlation between miR21 and *RECK* levels (r_s_ = −0.2861, *p* = 0.2351, Figure 2B).

**Figure 3:**
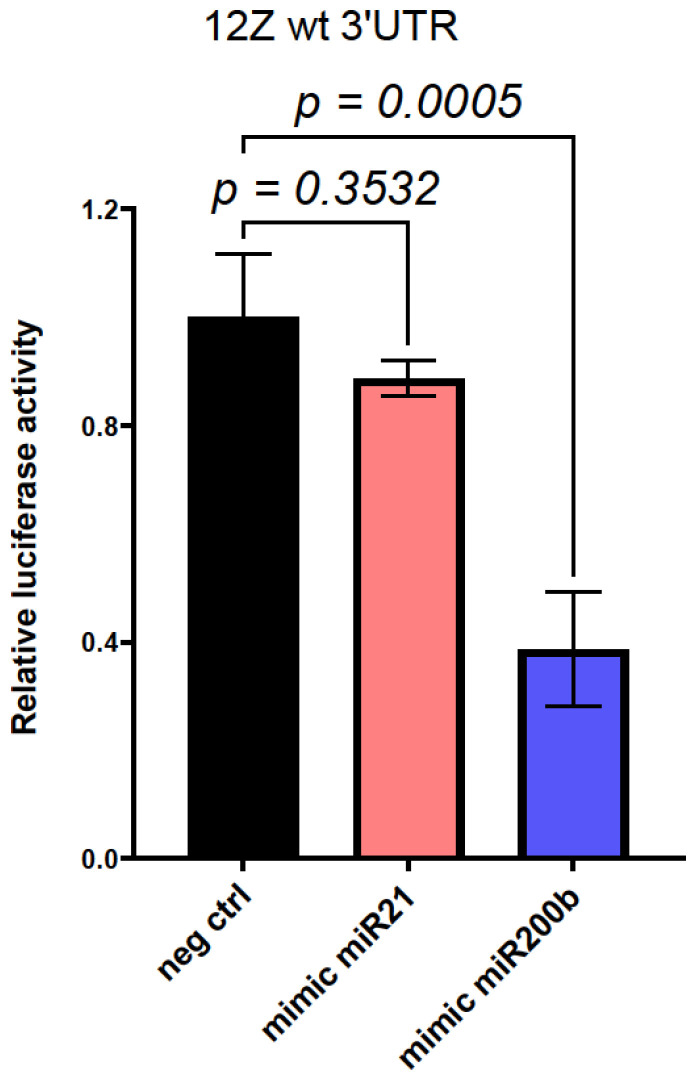
Effect of miR21 and miR200b on activity of wt 3′UTR of *RECK* gene in 12Z cells. 12Z cells were co-transfected with the *RECK* 3′UTR-pMIR plasmid together with microRNA mimics for miR21, miR200b, or negative control miR. Luciferase activity was measured 24 h after transfection. One-way ANOVA with Tukey’s post-analysis test was used to evaluate the statistical significance of the results. Graphs depict mean values obtained in 4 independent experiments, each conducted in triplicate.

To verify whether miR200b and miR21 MREs in *RECK*-3′UTR are active in cells of endometrial origin, a construct carrying the luciferase gene fused with *RECK*-3′UTR and controlled by CMV promoter was generated as described in detail in the Section 4. The resulting *RECK*-3′UTR-PMIR plasmid was introduced into the endometriotic cell line 12Z together with particles mimicking miR200b and miR21 (microRNA mimics). As shown in Figure 3, the introduction of the miR200b mimic strongly reduced the activity of *RECK*-3′UTR in the endometriotic 12Z cell line, whereas the co-expression of miR21 mimics did not affect *RECK*-3′UTR activity. 

**Figure 4:**
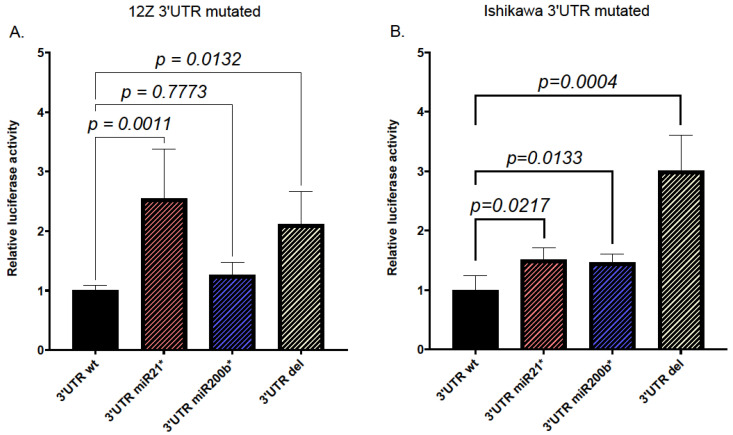
Effect of the disruption of the miR21 and miR200b MRE sites on the activity of the 3′UTR of the *RECK* gene. 12Z (**A**) and Ishikawa cell lines (**B**) were transfected with wt *RECK* 3′UTR-pMIR or its mutants and luciferase activity was measured 24 h after transfection. The constructs mutated at the sites of putative recognition by the particular microRNA are denoted by an asterisk and microRNA name. The construct devoid of *RECK*-3′UTR (3′UTR del) served as a positive control. Relative luciferase activity is shown as a fold of the wt control. Graphs depict mean values obtained in 4 experiments, each conducted in triplicate. Error bars represent S.D. One-way ANOVA with Tukey’s post-analysis test was used to evaluate the statistical significance of the results.

To determine *RECK*-3′UTR response to endogenous microRNAs, the previously identified MREs in *RECK*-3′UTR-PMIR were mutated, and the activity of the mutants was evaluated in the 12Z cell line and Ishikawa cell line. The deletion of MREs for miR21 in *RECK*-3′UTR resulted in a significant increase in luciferase activity in both 12Z and Ishikawa cells (Figure 4). On the other hand, the deletion of miR200b MRE upregulated luciferase activity in Ishikawa cells (Figure 4B) but had no effect in 12Z cells (Figure 4A).

**Figure 5:**
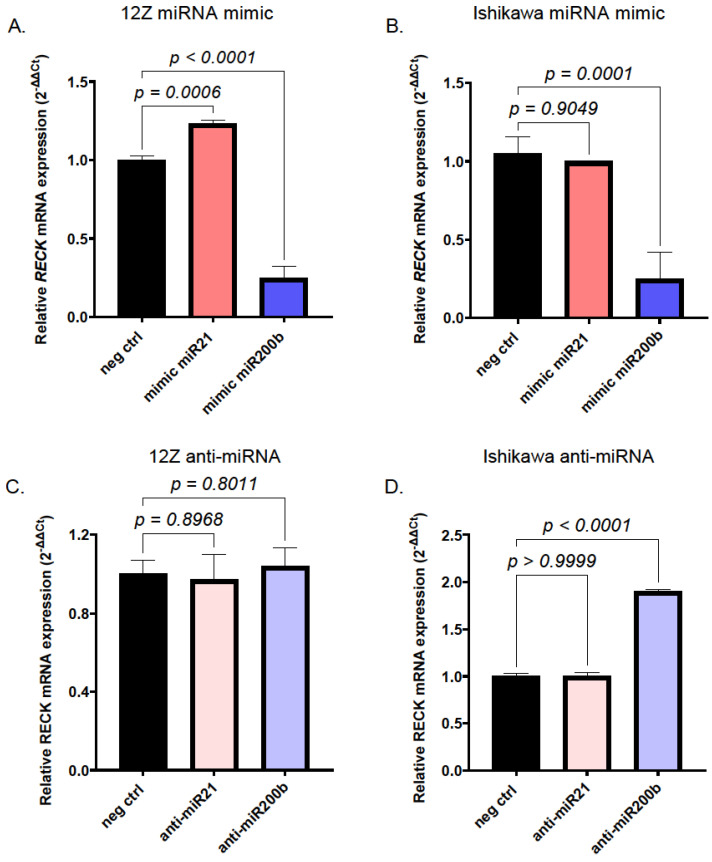
Effect of miR200b and miR21 on expression of endogenous *RECK* gene. Effect of transfection with miR200b or miR21 mimic oligonucleotides on *RECK* mRNA in 12Z (**A**) and Ishikawa (**B**) cells. Effect of transfection with anti-miR200b and anti-miR21 oligonucleotides on *RECK* mRNA in 12Z (**C**) and Ishikawa (**D**) cells. Effect of miR overexpression or inhibition on relative *RECK* mRNA expression was determined with qRT-PCR. Data are expressed as fold of control, e.g., *RECK* mRNA level in cells transfected with negative control for miR mimic or anti-miR particles, respectively. Graphs present mean values ± SD obtained in 3 independent experiments. One-way ANOVA with Tukey’s post-analysis test was used to evaluate the statistical significance of the results.

To validate the results obtained in experiments using the *RECK*-3′UTR constructs, we evaluated the effect of miR200b and miR21 on the expression of the endogenous *RECK* gene. For this purpose, mimic miR200b and miR21 oligonucleotides were introduced into 12Z and Ishikawa cell lines. An overexpression of the miR200 mimic was able to diminish *RECK* gene expression in both cell lines (Figure 5A,B). The overexpression of the miR21 mimic slightly increased *RECK* levels in the 12Z cell line (Figure 5A) and had no effect on *RECK* transcript levels in the Ishikawa cell line (Figure 5B). To evaluate the effects of the inhibition of endogenous miR200b and miR21, the cells were transfected with their respective antagomiRs (anti-miRs). *RECK* expression in 12Z cells appeared to be insensitive to both anti-miRs (Figure 5C). On the other hand, the expression of *RECK* was increased two-fold in Ishikawa cell line upon transfection with anti-miRs targeting miR200b, while antago*miRs* targeting miR21 had no effect (Figure 5D). 

**Figure 6:**
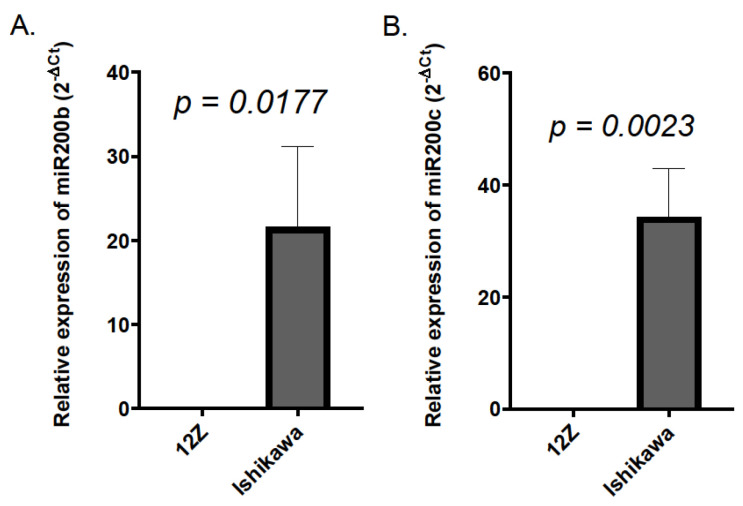
The basal levels of miR200b (**A**) and miR200c (**B**) in Ishikawa and 12Z cell lines. Data are expressed as 2^−ΔCt^. Graphs present mean values obtained in 3 experiments. Error bars represent S.D. The Mann–Whitney U-test was used to evaluate the statistical significance of the results.

To uncover the reason for the observed difference between the data obtained in 12Z and Ishikawa cell lines regarding the effect of mimic and anti-miR particles, the basal levels of the two miR200 transcripts, i.e., miR200b and miR200c, were compared in both investigated cell lines. The measurement revealed that the level of miR200b was almost 200 times higher in the Ishikawa cell line than in 12Z cells (the 2^−ΔCt^ value was 21.61 ± 9.57 and 0.11 ± 0.057 for Ishikawa cells and 12Z cells, respectively, Figure 6A). The level of miR200c in Ishikawa cells exceeded that detected in 12Z cells more than 1000-fold (the 2^−ΔCt^ value was 34.29 ± 8.6 and 0.02 ± 0.01 for Ishikawa cells and 12Z cells, respectively, Figure 6B). 

## 3. Discussion

Our study demonstrates that *RECK* expression is higher in endometriotic tissue when compared to the control endometrium, e.g., derived from women without endometriosis. Interestingly, the levels of other TIMPs’ gene expression, especially those that comprise long 3′UTR and are known to be repressed by micro-RNAs, were reported to be altered in ectopic endometriotic tissue (summarized in [21]). Thus, our present observation confirms the complexity of tissue remodeling networks in endometriosis.

The bioinformatic analysis has revealed that *RECK* 3′UTR displayed one MRE for miR21, one for miR181a, one MRE for miR182, and two MREs for the miR200bc family. However, we did not reveal any differences in the expression of miR181a and miR182 in the endometriotic tissue as compared to the control endometrium. Therefore, they were excluded from further investigations. On the other hand, the levels of miR200b and miR21 were demonstrated to be significantly reduced in the endometriotic tissue. A decreased expression of miR200a, miR200b, and miR200c in endometriotic tissue (ectopic endometrium) when compared to the control endometrium was previously detected employing microRNA microarray [62,63], and RNA-sequencing analyses [64], followed by hit validation with RT-PCR. However, none of the global miRnome analyses demonstrated valid differences in miR21 levels in endometriotic tissue vs. control endometrium.

The direct targeting of *RECK* by miR200 has been documented in colorectal cancer specimens and cell lines [48], lung cancer [49], and bladder cancer [50]. On the other hand, the repressive effect of miR21 on *RECK* expression has been described in cancerous tissues or cells, e.g., glioma [51,52], oral cancer [53], gastric cancer [54], prostate cancer [55], and hepatocellular carcinoma [56,57], as well as non-transformed tissues and cells, like mesenchymal stem cells [58], cardiac fibroblasts [59], and renal tubules [60]. 

The Spearman correlation analysis of *RECK* mRNA levels vs. miR200b and miR21 indicated a significant inverse correlation between *RECK* and miR200b levels in patients’ specimens, whereas the absolute value of the correlation coefficient in *RECK* vs. miR21 analysis was much lower, and the results did not reach statistical significance. These observations suggest the involvement of miR200b in the reduction of *RECK* mRNA expression in endometriotic tissue and a probably less important role played by miR21. 

We further confirmed the role of miR200b in the negative regulation of *RECK* mRNA expression using a luciferase gene reporter assay. The assays employing the fusion construct of the *RECK* 3′UTR sequence and luciferase DNA showed that both miRNA types affected *RECK* 3′UTR stability. The specificity of this effect was confirmed by the disruption of MREs specific for particular miRNAs. As expected, the mutation of the *RECK* 3′UTR sequence that was previously shown to be targeted by miR21 [51,52] resulted in higher *RECK* 3′UTR activity. This result is intriguing given the observation that mimic and anti-miR21 had no effect on the expression of endogenous *RECK* in 12Z and Ishikawa cells. It should be stressed, however, that MREs for miR21 may be shared with other microRNAs, or mutation may influence the stability of the construct by some unknown mechanism(s). In conclusion, these results suggest that miR21 does not play a significant role in *RECK* mRNA regulation in endometriotic cells. 

Interestingly, upon miR200b inhibition, an increased *RECK* expression was evident only in Ishikawa cells, but not in 12Z cells. Similarly, the expression of the canonical targets of miR200b, namely ZEB1 and ZEB2 genes [44], was strongly induced in Ishikawa cells upon miR200b inhibition, but not in 12Z cells (unpublished results). The observed difference may be due to the relatively low level of endogenous miR200b in the 12Z cell line (Figure 6A,B). 12Z cells were isolated from endometriotic lesions [65]. Our present results (Figure 1C), and previously published data [62,63,64], indicate that miR200b expression is strongly decreased in endometriotic lesions when compared to control tissue. Thus, the 12Z cell line seems to replicate the pattern of expression of miR200b and its targets present in endometriotic tissue. Accordingly, a low level of miR200b in 12Z cells determines the EMT gene expression observed in this cell line [61]. On the other hand, it is worth acknowledging that miR200b expression is upregulated in endometrial cancer [66,67], and the vast difference in the expression of miR200 species between 12Z and Ishikawa cell lines may just demonstrate two opposite poles of the spectrum. Nevertheless, both cell lines share the same mechanism of the regulation of *RECK* expression, e.g., its suppression by the sufficient expression of miR200b, and minute effect following manipulation with miR21 levels. 

Previous functional analyses had indicated that the upregulation of miR200b in 12Z inhibits invasive growth in vitro [59]. While this phenotype was attributed to altered EMT, our novel finding of a targeting of *RECK* by this miRNA suggests that the inhibition of proteolytic activity may be an additional factor contributing to the anti-invasive properties of miR200b, worthy of further investigation.

In conclusion, our present results strongly imply that *RECK* mRNA expression is significantly increased in endometriotic lesions. It seems that this effect is due to downregulated miR200b; the mechanism of this downregulation remains, however, unclear. Similarly, it is not clear what is the meaning of the increased *RECK* expression in view of the pathogenesis of endometriosis. While endometriosis is characterized by a higher production of MMPs, followed by an increased invasiveness of endometriotic cells [12,21,22,23], an elevated *RECK* expression may represent a regulatory feedback mechanism, opening up interesting new search routes. 

## 4. Materials and Methods

### 4.1. Patients and Tissue Collection

This study was approved by the Bioethical Committee of the Medical University of Warsaw and Military Institute of Medicine (approval no. KB/223/2009 and 49/WIM/2011) and was conducted according to strict institutional guidelines in accordance with the 1975 Declaration of Helsinki. All patients gave informed written consent to the study.

The study group consisted of 14 patients with endometriosis and 8 control women who were diagnosed and donated material between January 2009 and June 2013 at the 1st Department of Obstetrics and Gynecology, Medical University of Warsaw, and the Department of Gynecology and Gynecological Oncology of the Military Institute of Medicine-National Research Institute in Warsaw. All women had regular menses, and none of them had a history of previous pelvic surgery, chronic systemic disease, or cancer. The patients were not subjected to any hormonal or immunomodulatory therapy for at least three months prior to the study. All samples from the control women and patients with endometriosis were collected in the mid-luteal phase of the menstrual cycle. The mid-luteal phase of the cycle was confirmed by ultrasound examination of the ovaries. Endometriotic tissue from ovarian endometriotic cysts was collected during laparoscopic surgery. Control tissue was derived from eutopic endometrium from women who were treated for other conditions and diagnosed as free from endometriosis. Demographical data of controls and patients are summarized in Table 1. 

### 4.2. Cell Culture

The effects of manipulation with microRNA mimics and microRNA inhibitors on *RECK* levels were tested in the endometriotic cell line 12Z [65] and endometrial adenocarcinoma cell line Ishikawa, purchased from the American Tissue Culture Collection (Gaithersburg, MD, USA). The cell culture medium was composed of DMEM-F12 (Dulbecco Modified Essential Medium with F-12 supplement) with Glutamax, 1% Penicillin-Streptomycin Solution, and FBS (Foetal Bovine Serum) at 10% and 5% concentrations for the 12Z and Ishikawa cells, respectively. All the above reagents were purchased from Thermo Fisher Scientific (Waltham, MA, USA). Cells were cultured in a 5% CO_2_ incubator at 37 °C and split with the use of Accutase (Merck KGaA, Darmstadt, Germany) before full confluency was reached.

### 4.3. RNA Isolation from Donor Tissue and Cell Cultures

Total RNA was isolated from frozen biopsies of donor tissue using TRIzol reagent (Thermo Fisher Scientific). RNA from cultured cell lines was purified with a NucleoSpin miRNA kit from Macherey-Nagel GmbH and Co., KG (Düren, Germany). Ten µg of Carrier RNA from the same manufacturer was added during the isolation procedure to prevent the loss of small RNA. 

### 4.4. Quantitative Reverse Transcription Polymerase Chain Reaction (qRT-PCR)

To obtain cDNA from mRNA, reverse transcription was performed in Eppendorf MasterCycler Gradient (Eppendorf AG, Hamburg, Germany) using a Reverse Transcription System (Promega Corp., Madison, WI, USA). In order to synthesize cDNA from microRNA, a TaqMan^®^ MicroRNA Reverse Transcription Kit was employed, together with dedicated primers from the TaqMan^®^ microRNA Assay. The IDs of the assays used for the evaluation of miRNA expression with qRT-PCR were as follows: 001095 for RNU43, 000397 for miR21, 000480 for miR181a, 000597 for miR182, 002251 for miR200b, and 002300 for miR200c (Thermo Fisher Scientific).

The expression of the *RECK* gene and endogenous control gene *GAPDH* was evaluated using specific TaqMan^®^ gene expression assays with the IDs Hs01019179_m1 and Hs02758991_g1, respectively (Thermo Fisher Scientific), according to the instructions provided by the manufacturer. Reactions (10 μL) were run in triplicate on the 7500 Fast Real-Time PCR System (Applied Biosystems, Woolston, Cheshire, UK) using TaqMan^TM^ Fast Advanced Master Mix (Thermo Fisher Scientific). To compensate for differences in total mRNA amounts, the results were normalized to *GAPDH* expression levels. To determine the level of microRNA expression, the TaqMan^®^ microRNA Assays described above were employed; the rest of the reagents and methods were the same as for the measurement of mRNA expression. Here, *RNU43* served as an endogenous control for the qPCR measuring microRNA expression. Gene expression data are presented as RQ (Relative Quantification) or 2^−ΔCt^.

### 4.5. Cloning and Mutagenesis of 3′UTR of *RECK* Gene

**Figure 7:**
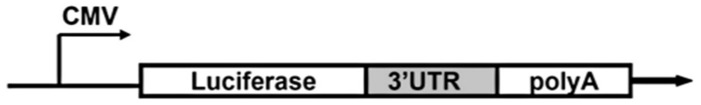
The diagram of the *RECK* 3′UTR- luciferase insert.

The 3′UTR of the *RECK* gene was cloned from the gDNA of a healthy volunteer. In brief, the 3′UTR *RECK* sequence (ENSG00000122707) was amplified by PCR using Qiagene Hot Start Plus polymerase (Promega Corp.) with sense 5′-TCTGAGCTCATCATTTCCCAGGTACAG-3′ and antisense 5′-GCAGCGGCCGCAGCTCACCTAAGGG-3′ primers containing *SacI* and *NotI* restriction sites, respectively. The resulting 1621bp long amplicon was cloned into a pJet 1.2 vector using the CloneJET PCR Cloning Kit (Fermentas Inc., Glen Burnie, MD, USA) according to the manufacturer protocol. Minipreps from positive clones were digested with *SacI* and *NotI* restriction enzymes; the released inserts were isolated from agarose gel, purified, and subcloned into the pMIRreport vector, developed to evaluate the activity of UTRs derived from genes of interest (Ambion, Austin, TX, USA). The resulting colonies were screened by PCR to select clones displaying the correct insert orientation. The thereby obtained *RECK*-3′UTR-pMIR construct of 7970bp length was purified and sequenced. The resulting construct carried the luciferase coding sequence under the control of CMV promoter and *RECK* 3′UTR (Figure 7). 

**Figure 8:**
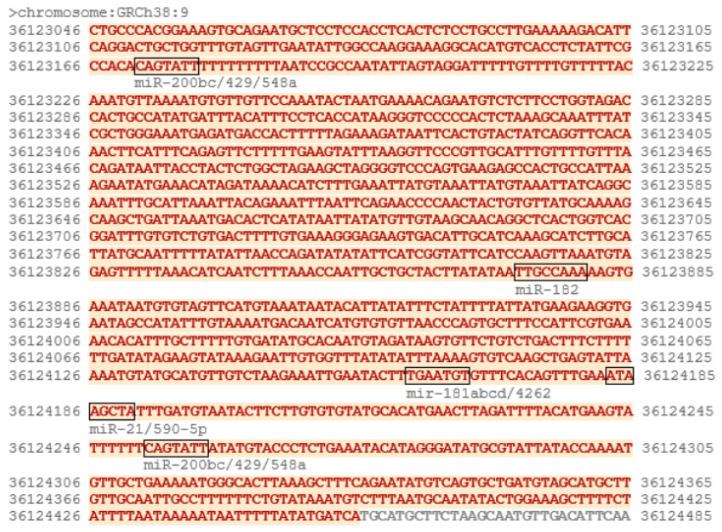
Putative MREs in human *RECK* 3′UTR were predicted using TargetScan 8.0. The 3′UTR sequence is shown in red, with the predicted sites recognized by specific miRNA species enclosed in boxes.

The TargetScan ver. 8.0 tool (https://www.targetscan.org/vert_80/, accessed on 10 October 2022) was used to detect the sequences in *RECK*-3′UTR that were potentially recognized by microRNA species (MRE—micro-RNA Responsive Elements). The results of the analysis are shown in Figure 8. 

Based on the above analysis, the plasmids carrying mutations in the 3′UTR of the *RECK* gene that render them unrecognizable for miR21, and miR200 were generated on a wild-type (wt) *RECK* 3′UTR-pMIR template. Guanine to adenine mutagenesis was carried out using the QuickChange II Site-Directed Mutagenesis Kit (Agilent Technologies, Santa Clara, CA, USA) according to the manufacturer’s instructions. The primer sets for mutagenesis and the names of all mutants are listed in Table 2. The outcome of mutagenesis was confirmed by sequencing. Since 2 sites in *RECK*-3′UTR may be targeted by miR200, both were mutated sequentially, yielding a double mutant. 

### 4.6. Luciferase Reporter Assay 

Ishikawa and 12Z cells were transfected in triplicate with *RECK*-3′UTR-pMIR plasmids using Lipofectamine 2000 (Thermo Fisher Scientific) according to the manufacturer’s protocol. Each transfection was performed with 0.8 µg of the respective pMIR construct and 0.05 µg of the pRL-TK vector encoding *Renilla* luciferase (Promega Corp.), which was used to normalize the assay. The cells were harvested on the following day and assayed using reagents of the Dual-Luciferase Reporter Assay System (Promega Corp.) and the FLUOstar Omega luminometer (BMG Labtech, Offenburg, Germany). The background luminescence of the lysate from untransfected cells was subtracted from the sample’s luminescence. The results were expressed as a ratio between the luminescence resulting from pMIR and pRL-TK expression.

### 4.7. Transfection with microRNA Mimics and Inhibitors

Twenty-four hours before transfection, 12Z and Ishikawa cells were seeded onto 12-well plates at a density of 100,000 and 70,000 cells per well, respectively.

Oligo RNAs (Thermo Fisher Scientific) used for the transfection are listed in Table 3. The transfection was performed with the use of Lipofectamine RNAiMAX Reagent (Thermo Fisher Scientific) according to the manufacturer’s instruction, in an antibiotic-free growth medium. Briefly, to the cells growing on one well of a 12-well plate, a mixture of 6 µL of RNAiMAX and 20 pmol of microRNA mimic or inhibitor was added. Cells were left with the transfection mix for 48 h, e.g., until the moment of RNA collection. 

### 4.8. Statistical Analysis

GraphPad Prism v9.3.1 for Windows (GraphPad Software, San Diego, CA, USA) was used for the statistical analysis of the data. Whenever two groups were compared, and did not pass the normality test, a Mann–Whitney U-test was applied. When more than 2 groups were analyzed, a one-way ANOVA followed by Tukey’s post-analysis test was used. To evaluate a possible correlation between data sets, the Spearman correlation coefficient was computed. The difference between experimental conditions was considered significant if *p* < 0.05.

## 5. Conclusions

The present study demonstrates that the expression of the *RECK* gene is elevated in endometriotic lesions when compared to control endometrium from women without endometriosis. The increase in *RECK* gene mRNA levels in patient samples was accompanied by a reduction in miR200b levels. The expression of both genes in clinical samples showed a strong inverse correlation. The suppressive effect of miR200b on *RECK* gene expression was confirmed in the 12Z cell line, derived from a peritoneal endometriotic lesion, and the Ishikawa cell line, originating from endometrial adenocarcinoma. First, we show that the 3′UTR of the *RECK* gene is a direct target of miR200b. Second, we demonstrate that miR200b downregulates the expression of endogenous *RECK* mRNA in the investigated cell lines. The demonstrated effect of miR200b on *RECK* gene expression, together with the altered expression of both genes in endometrioma, may help to recognize the role played by the inhibitors of metalloproteases in the pathogenesis of endometriosis. 

## Figures and Tables

**Table 1:** Characteristics of patients with endometriosis and control subjects.

		Control	Endometriosis
Number of cases (N)		8	14
Age, years (mean ± SD)		35.2 ± 6.40	31.9 ± 5.53 *
rASRM	I/II (minimal/mild)	NA	2 (14.3%)
	III/IV (moderate/severe)	NA	12 (85.7%)
Lesion localization	Ovarian	NA	14 (100%)
	Peritoneal	NA	12 (85.7%)
	Both	NA	12 (85.7%)

NA, not applicable; *p*-values were calculated by Student’s *t*-test. * Different from the control group at *p* = 0.02.

**Table 2:** Primers used for *RECK*-3′UTR construct cloning and mutagenesis. The asterisk indicates that an MRE putatively targeted by a particular miR has been mutated.

Primer Name	Primer Sequence	Construct Name
	Cloning	
FSacI	5′-TCTGAGCTCATCATTTCCCAGGTACAG-3′	wt *RECK*-3′UTR-pMIR
RNotI	5′-GCAGCGGCCGCAGCTCACCTAAGGG-3′	
	Mutagenesis	
3Re21F	5′-GTGTTTCACAGTTTGAAATATGCTATTTGATGTAATACTTC-3′	*RECK*-3′UTR-pMIRmiR21 *
3Re21R	5′-GAAGTATTACATCAAATAGCATATTTCAAACTGTGAAACAC-3′	
3Re200F1	5′-CCTCTATTCGCCACACAGAATTTTTTTTTTTAATCC-3′	*RECK*-3′UTR-pMIRmiR200×2 *
3Re200R1	5′-GGATTAAAAAAAAAAATTCTGTGTGGCGAATAGAGG-3′	
3Re200F2	5′-CATGAAGTATTTTTTCAGAATTATATGTACCCTCTG-3′	
3Re200R2	5′-CAGAGGGTACATATAATTCTGAAAAAATACTTCATG-3′	

**Table 3:** List of microRNA mimics and microRNA inhibitors used for transfection.

Type of Oligo Rna	Assay ID
*miR*Vana^®^ microRNA mimic, hsa-miR21-3p	MC12979
*miR*Vana^®^ microRNA mimic, hsa-miR200b-3p	MC10492
*miR*Vana™ microRNA Mimic, Negative Control #1	---
*miR*Vana^®^ microRNA inhibitor, hsa-miR21-3p	MH12979
*miR*Vana^®^ microRNA inhibitor, hsa-miR200b-3p	MH10492
*miR*Vana™ microRNA Inhibitor, Negative Control #1	---

## Data Availability

The data and their interpretation are contained in the research article.
